# Fibrillar extracellular matrix produced by pericyte‐like cells facilitates glioma cell dissemination

**DOI:** 10.1111/bpa.13265

**Published:** 2024-05-05

**Authors:** Petr Vymola, Elena Garcia‐Borja, Jakub Cervenka, Eva Balaziova, Barbora Vymolova, Jana Veprkova, Petr Vodicka, Helena Skalnikova, Robert Tomas, David Netuka, Petr Busek, Aleksi Sedo

**Affiliations:** ^1^ Laboratory of Cancer Cell Biology, Institute of Biochemistry and Experimental Oncology, First Faculty of Medicine Charles University Prague Czech Republic; ^2^ Laboratory of Applied Proteome Analyses, Research Center PIGMOD Institute of Animal Physiology and Genetics of the Czech Academy of Sciences Liběchov Czech Republic; ^3^ Laboratory of proteomics, Institute of Biochemistry and Experimental Oncology, First Faculty of Medicine Charles University Prague Czech Republic; ^4^ Department of Neurosurgery Na Homolce Hospital Prague Czech Republic; ^5^ Department of Neurosurgery and Neurooncology, First Faculty of Medicine Charles University and Military University Hospital Prague Czech Republic

**Keywords:** collagen type I, extracellular matrix proteins, fibronectin, glioblastoma, pericytes, proteomics

## Abstract

Gliomagenesis induces profound changes in the composition of the extracellular matrix (ECM) of the brain. In this study, we identified a cellular population responsible for the increased deposition of collagen I and fibronectin in glioblastoma. Elevated levels of the fibrillar proteins collagen I and fibronectin were associated with the expression of fibroblast activation protein (FAP), which is predominantly found in pericyte‐like cells in glioblastoma. FAP+ pericyte‐like cells were present in regions rich in collagen I and fibronectin in biopsy material and produced substantially more collagen I and fibronectin in vitro compared to other cell types found in the GBM microenvironment. Using mass spectrometry, we demonstrated that 3D matrices produced by FAP+ pericyte‐like cells are rich in collagen I and fibronectin and contain several basement membrane proteins. This expression pattern differed markedly from glioma cells. Finally, we have shown that ECM produced by FAP+ pericyte‐like cells enhances the migration of glioma cells including glioma stem‐like cells, promotes their adhesion, and activates focal adhesion kinase (FAK) signaling. Taken together, our findings establish FAP+ pericyte‐like cells as crucial producers of a complex ECM rich in collagen I and fibronectin, facilitating the dissemination of glioma cells through FAK activation.

## Graphical abstract

FAP+ pericyte‐like cells drive the deposition of fibrillar extracellular matrix proteins such as collagen I and fibronectin in glioblastoma, facilitating glioma cell dissemination and activation of focal adhesion kinase.

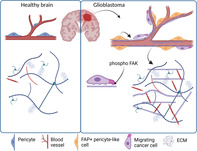

## 1 INTRODUCTION

Glioblastoma (GBM) is the most common primary brain tumor characterized by a poor prognosis with a median survival of 12–15 months [1]. One of the hallmarks of GBM is its ability to rapidly infiltrate and invade the surrounding brain tissue [2, 3], which prevents effective surgical removal and limits the possibility of cure. The infiltration into the surrounding tissue predominantly takes place along blood vessels [4, 5, 6] in regions referred to as perivascular niche (PVN). PVN consists of highly heterogenous cellular and acellular components [7, 8]. It is resided by endothelial cells, pericytes, macrophages, and glioma stem‐like cells (GSCs). Additionally, it contains an extracellular matrix (ECM) [5, 9, 10] composed of fibrillar ECM proteins including collagen I (COLI) and fibronectin (FN1) [11, 12, 13].

Gliomagenesis is associated with changes in the quantity and composition of fibrillar ECM proteins. It was previously described that alterations in the architecture of COLI support tumor progression and are associated with shorter overall survival in GBM [14, 15, 16], possibly because COLI forms a rigid environment that guides glioma cell invasion along blood vessels [17, 18]. FN1 is one of the most frequently upregulated ECM proteins in various solid tumors, including GBM. In perivascular regions, FN1 promotes the differentiation of GSCs, potentially leading to a more invasive phenotype of these cells and contributing to poor prognosis of the patients [12, 19, 20]. Thus, COLI and FN1 represent important molecules involved in GBM progression. Nevertheless, their cellular source in GBM remains largely unknown. The interaction of ECM proteins with cell receptors, such as integrins, initiates intracellular signaling cascades including activation of a focal adhesion kinase (FAK) [21, 22]. This non‐receptor tyrosine kinase promotes the malignant phenotype of glioma cells by enhancing their proliferation, migration, and invasion [23, 24, 25].

We and others have identified pericyte‐like mesenchymal cells that express the serine protease fibroblast activation protein (FAP) as one of the stromal components that may be associated with worse survival of GBM patients [10, 26, 27]. Recently, we have demonstrated that this cellular population localized in the PVN plays an important role in GBM progression by promoting angiogenesis, migration, and proliferation of cancer cells by soluble mediators [27]. In this study, we identified another potential mechanism by which this subpopulation may contribute to gliomagenesis. We demonstrate that FAP+ pericyte‐like cells are the main producers of fibrillar ECM, such as COLI and FN1, in GBM. Our results further indicate that this ECM promotes glioma cell migration, adhesion, and activation of FAK signaling.

## 2 RESULTS

### 2.1 Collagen I and fibronectin levels are associated with FAP expression in GBM

**FIGURE 1:**
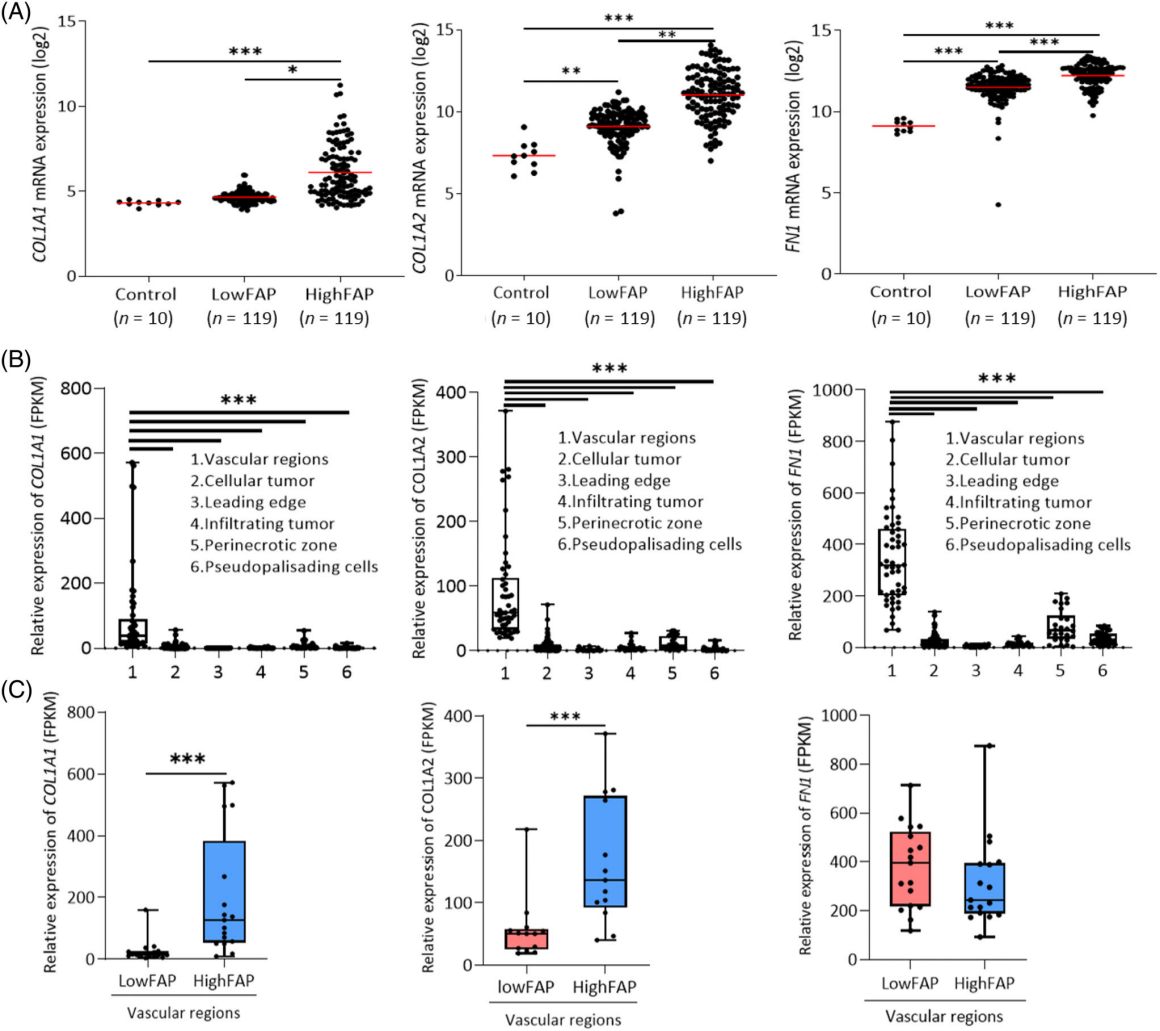
Expression of COLI genes and FN1 in GBM is associated with high expression of FAP. (A) TCGA database‐based bioinformatic analysis of COL1A1, COL1A2, and FN1 expression in control, non‐tumorous brain tissue (*n* = 10) and GBMs with high (*n* = 119, upper tercile) and low (*n* = 119, lower tercile) expression of FAP. *p <0.05 **p <0.01, ***p <0.001, Kruskal–Wallis test. (B) Expression of COL1A1, COL1A2, and FN1 in distinct regions of GBM. Box–10th to 90th percentile, whiskers—min to max values, dots—original data, line—mean, ***p <0.001, ANOVA, Tukey's multiple comparison test. (C) Relative expression of COL1A1, COL1A2, and FN1 in vascular regions of GBM with high (*n* = 17, upper tercile) and low (*n* = 17, lower tercile) expression of FAP. Box—10th to 90th percentile, line—mean, whiskers—min‐max values, dots—raw data, ***p <0.001, Mann–Whitney U test.

Expression of collagen I (*COL1A1*, *COL1A2*) and *FN1* was higher in GBM than in non‐tumorous brain tissue according to The Cancer Genome Atlas (TCGA) Database (Supplementary Figure 1). To investigate the relation between *FAP*, COLI (*COL1A1*, *COL1A2*), and *FN1* expression in GBM, we divided 357 primary IDH‐wild type GBMs into terciles according to the expression of *FAP* and included the upper (*HighFAP*, *n* = 119) and the lower (*LowFAP*, *n* = 119) terciles in the following analyses. The highest expression of both COLI genes and *FN1* was in *HighFAP* GBMs (Figure 1A). There was no significant difference in *COL1A1* expression between *LowFAP GBMs* and non‐tumorous brain (Figure 1A).

To further support the association between the expression of *FAP*, *COL1A1*, *COL1A2*, and *FN1*, we analyzed the transcriptomic data from histologically defined GBM regions available in the Ivy GBM Atlas [28]. As shown in Figure 1B, the expression of *COL1A1*, *COL1A2*, *and FN1* was significantly higher in vascular regions (“hyperplastic blood vessels” and “microvascular proliferation”) in comparison with parenchymal areas of GBM. In addition, the expression of *COL1A1* and *COL1A2* was 20–30‐fold higher in vascular regions rich in FAP (upper tercile, *n* = 17 vs. lower tercile, *n* = 17, Figure 1C). *FN1* expression was comparable in vascular regions with high and low FAP expression (Figure 1C).

**FIGURE 2:**
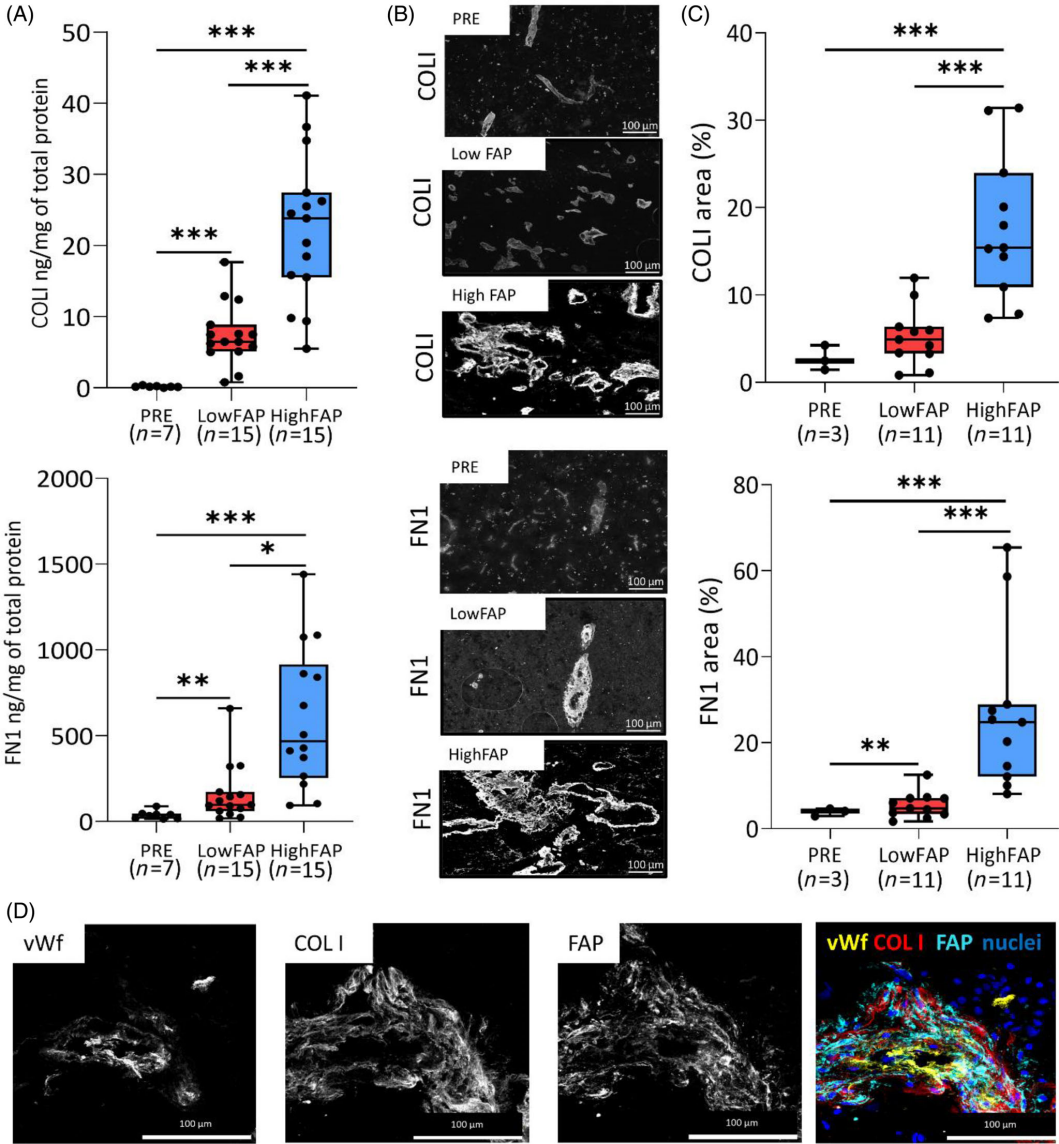
COLI and FN1 are expressed by FAP+ pericyte‐like cells in perivascular areas in GBM. A) COLI and FN1 concentration determined by ELISA in PRE (*n* = 7), LowFAP (*n* = 15), and HighFAP (*n* = 15) GBMs. Box–10th to 90th percentile, whiskers–min to max values, dots–original data, line–mean, *p <0.05 **p <0.01, ***p <0.001, Kruskal–Wallis test. (B) Representative immunofluorescence images of COLI and FN1 in PRE, LowFAP, and HighFAP GBMs. (C) Percentage of area covered by COLI and FN1 in PRE (*n* = 3), LowFAP (*n* = 11), and HighFAP (*n* = 11) GBMs. Box—10th to 90th percentile, line—mean, whiskers—min‐max values, dots—raw data, ***p < 0.0001, Kruskal–Wallis test. (D) A representative image of perivascularly localized collagen I (COLI) in close vicinity of FAP expressing cells; expression of von Willebrand factor (vWf) was used to identify endothelial cells.

We employed ELISA and immunohistochemistry to validate these findings at the protein level. Based on our previous study [29], GBM samples with high and low FAP expression (upper tercile, median 5.19 ng/mg of total protein—HighFAP and lower tercile, median 1.78 ng/mg of total protein—LowFAP, respectively) were selected. Pharmacoresistant epilepsy (PRE) samples were used as a control non‐tumorous brain tissue [29]. ELISA analyses confirmed that COLI and FN1 are more expressed in GBM than in non‐tumorous human brain (PRE). In addition, the concentration of COLI was three times higher in HighFAP GBM tissues compared to LowFAP and more than a hundred times higher than in PRE (Figure 2A). Similarly, FN1 expression was three times higher in HighFAP compared to LowFAP GBMs and more than 15 times higher than in PRE (Figure 2A). Using immunohistochemistry in an independent set of HighFAP and LowFAP GBMs (Figure 2B), we confirmed that the area covered by COLI and FN1 in HighFAP GBMs was higher than in LowFAP tumors and PRE (Figure 2C). Finally, immunohistochemistry revealed that COLI and FN1 were abundantly present in the PVN in the vicinity of FAP+ pericyte‐like cells (HighFAP, *n* = 5; LowFAP, *n* = 5) (Figure 2D). COLI and FN1 expression was not detected within the parenchyma of GBM (Supplementary Figure 2). Overall, these data show that the expression of COLI and FN1 on both transcriptomic and protein levels is associated with high expression of FAP in GBM and that both proteins are almost exclusively localized in perivascular areas resided by FAP+ pericyte‐like cells.

### 2.2 Collagen I and fibronectin are expressed by FAP+ pericyte‐like cells in vitro

**FIGURE 3:**
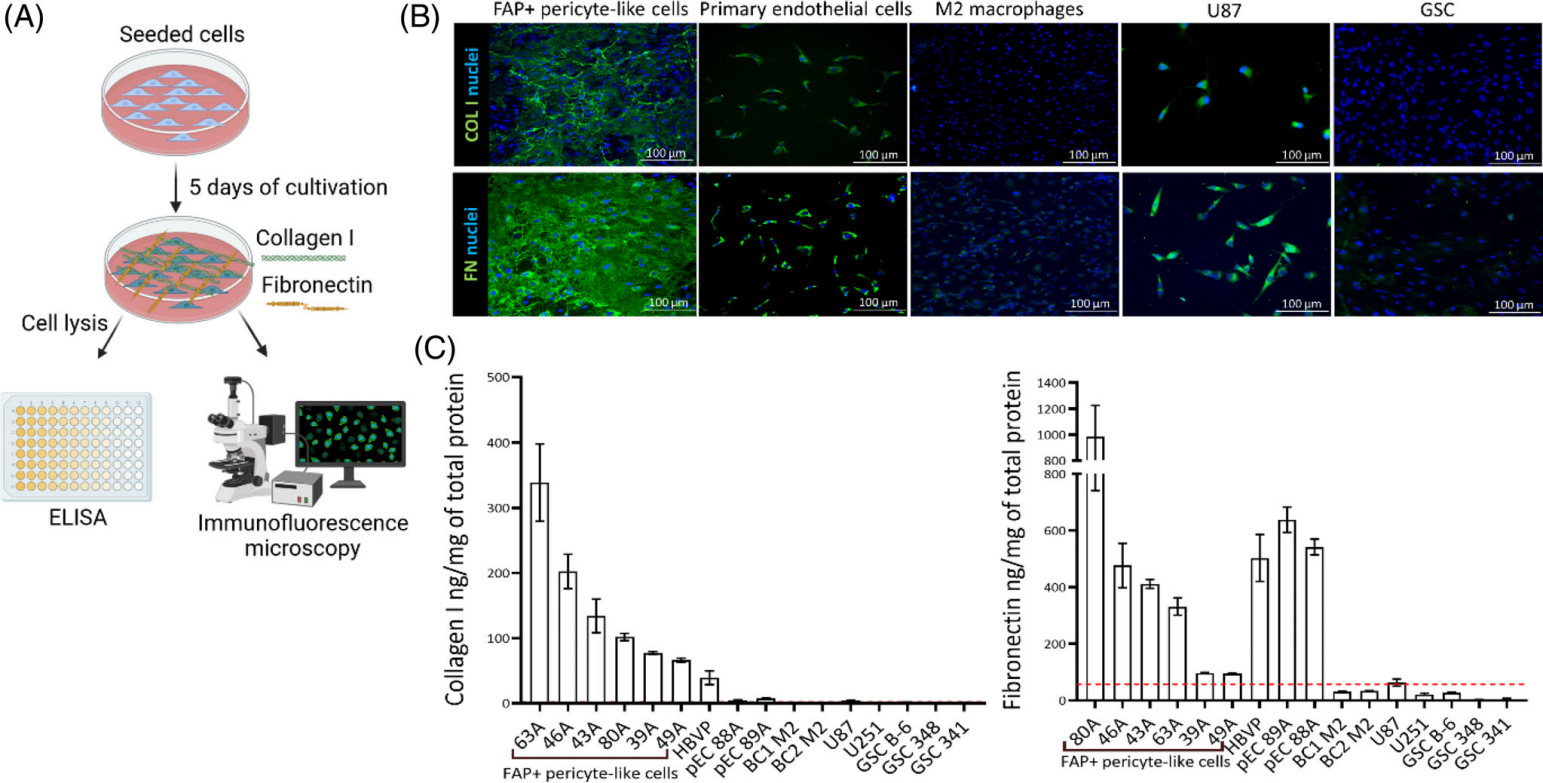
COLI and FN are expressed by FAP+ pericyte‐like cells in vitro. (A) A schema of experimental design. Created in Biorender.com. (B) Immunocytochemical detection and (C) quantification by ELISA of COLI and FN in cell types present in GBM. HBVP—human brain vascular pericytes, pEC—primary endothelial cells derived from GBM, BC1/2M2—primary M2 macrophages derived from monocytes, GSC—glioma stem‐like cells. Results are presented as mean ± SD from four parallel cell culture replicates, each measured in triplicate.

To assess whether FAP+ pericyte‐like cells significantly contribute to the production of fibrillar ECM in GBM, we analyzed COLI and FN1 expression in these cells, in primary endothelial cells derived from freshly resected GBM tissues [27], as well as in glioma cells (U87, U251, GSCs), primary M2 macrophages, and human brain vascular pericytes (HBVP) (Figure 3A). Immunofluorescence staining revealed COLI and FN1 immunopositivity in FAP+ pericyte‐like cells, HBVP, primary endothelial cells, and U87 (Figure 3B). To validate the immunostaining findings, we performed ELISA using cell lysates. ELISA confirmed the highest expression of COLI in FAP+ pericyte‐like cell cultures (*n* = 6, mean expression ranging from 66.3 to 338.7 ng/mg of total protein), compared to HBVP (39.13 ng/mg of total protein), endothelial cells derived from GBM (*n* = 2, pEC88A 4.3 ng/mg of total protein and pEC89A 7.5 ng/mg of total protein) and by U87 (3.8 ng/mg of total protein). No detectable COLI expression was observed in U251, GSCs (*n* = 3), and primary M2 macrophages (*n* = 2). Similarly, high expression of FN1 was observed in FAP+ pericyte‐like cells (*n* = 6, mean expression ranging from 94.4 to 984 ng/mg of total protein), HBVP (504 ng/mg of total protein), and primary endothelial cells derived from GBM (*n* = 2, mean expression ranging from 542.1 to 631 ng/mg of total protein). Expression of FN1 was detectable in one of the serum‐cultured glioma cell lines (U87; 62.8 ng/mg of total protein), whereas U251, three GSC cultures, and primary M2 macrophages (*n* = 2) showed no detectable expression (Figure 3C).

### 2.3 FAP+ pericyte‐like cells produce 3D matrices rich in collagen I and fibronectin

**FIGURE 4:**
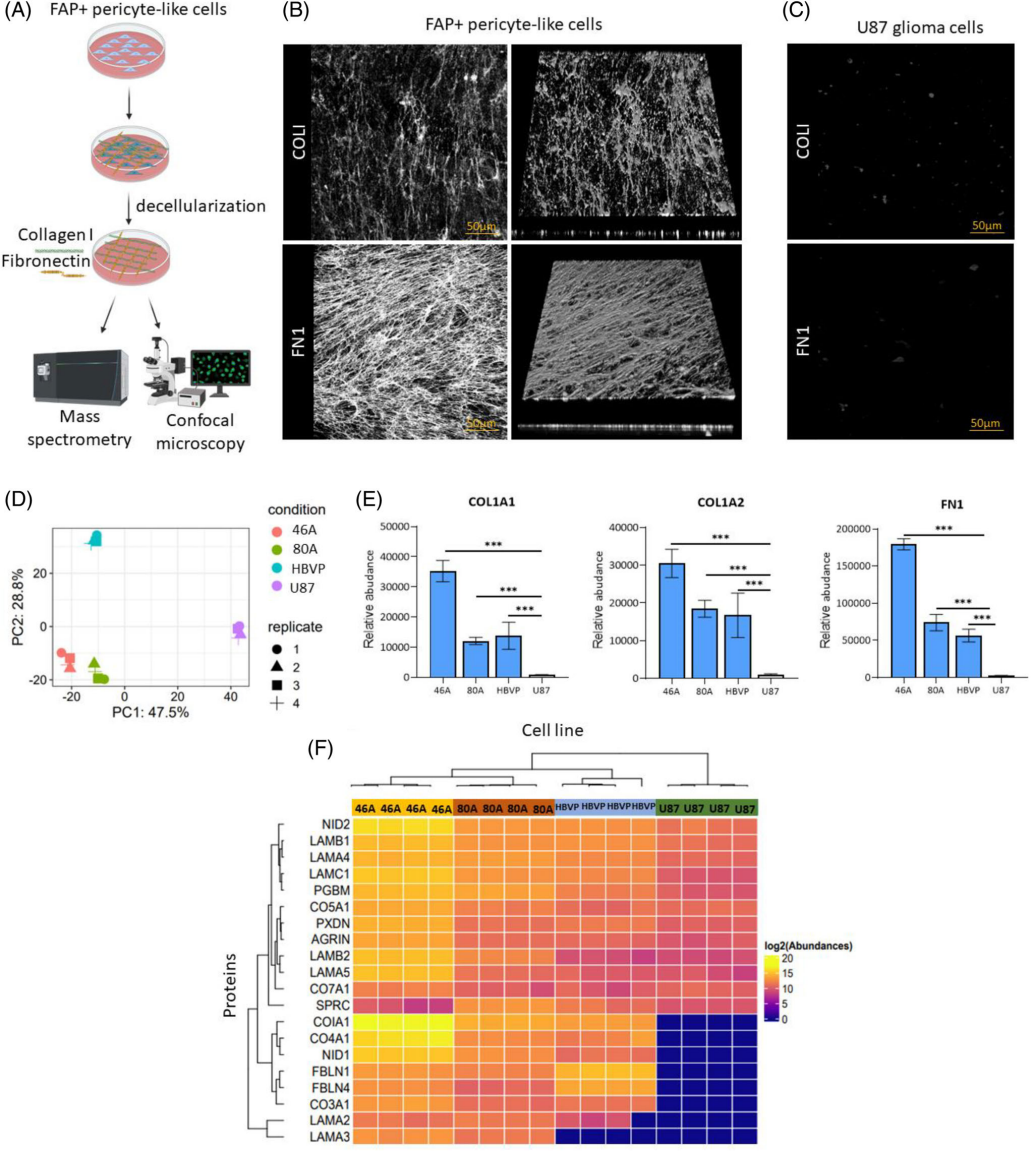
FAP+ pericyte‐like cells from human glioblastomas produce an ECM rich in collagen I, fibronectin, and essential proteins of basement membrane. (A) A schema of in vitro production of cell‐derived 3D matrices. Created in Biorender.com. (B) Immunodetection of COLI and FN1 in cell‐derived 3D matrices produced by FAP+ pericyte‐like cells (80A) and (C) U87 glioma cells. Representative confocal microscopy images (maximum intensity projection) and a 3D reconstruction. (D) Mass spectrometry analysis of matrisome proteins in cell‐derived 3D matrices produced by FAP+ pericyte‐like cells (80A, 46A), HBVP (human brain vascular pericytes), and U87 glioma cells. Principal component analysis (PCA), two‐dimension representation according to PC1 and PC2 based on the levels of 82 identified matrisome proteins. (E) Relative abundance of COL1A1, COL1A2, and FN1 in cell‐derived 3D matrices. Results are presented as mean ± SD from four replicates for each cell culture, ***p <0.001, ANOVA, Tukey's post hoc test. (F) Heatmap of relative protein abundance of basement membrane proteins in cell‐derived 3D matrices produced by FAP+ pericyte‐like cells (80A, 46A), HBVP, and U87 cells. Columns represent cell lines (quadruplicates) and rows represent expressed proteins as quantified by LC–MS/MS. Color in each tile represents the scaled log2 transformed abundance value.

To confirm the role of FAP+ pericyte‐like cells in fibrillar ECM deposition, we employed the 3D matrix production assay [30]. Using this assay, a layer of decellularized ECM can be produced in vitro and analyzed (Figure 4A). FAP+ pericyte‐like cells and HBVP formed a three‐dimensional (3D) network rich in COLI and FN1 as determined by immunohistochemistry (thickness of the COLI layer 8–12 μm for FAP+ pericyte‐like cells and 13.5 μm for HBVP; thickness of the FN1 layer 15.1–25.5 μm for FAP+ pericyte‐like cells and 26.7 μm for HBVP), while no COLI and FN1 were detectable in the decellularized ECM produced by U87 and U251 (Figure 4B, C). We next employed a quantitative mass spectrometry (MS) analysis to assess the global composition of the cell‐derived 3D matrix. Decellularized ECMs were prepared in four replicates from FAP+ pericyte‐like cells (46A and 80A), HBVP, U87, and U251 glioma cells; gelatin‐coated plates incubated without cells processed in the same way were used as controls. Protein concentration in all four ECM samples from U251 glioma cells and gelatin‐coated plates was below the detection limit of the BCA method (Supplementary Table 1) and intensities of MS‐measured peptides were notably lower than in the other samples. Only 62 proteins (Supplementary Table 2) were quantifiable in control, gelatin‐coated plates incubated without cells and the majority of these proteins was detected at low level. Because some of the detected proteins are components of blood plasma (e.g., FN1, apolipoprotein E, and prolactin‐inducible protein), we assume that all quantifiable proteins in control samples originated from the bovine serum in culture medium. Due to low number of quantifiable proteins, U251, and gelatin‐coated plate samples were excluded from further statistical analysis. Using the SWATH‐MS approach, we were able to quantify abundances of 3232 distinct proteins (Supplementary Table 3). Data analysis revealed that besides ECM proteins, intracellular proteins were also present in the samples of decellularized ECM, possibly reflecting the contamination by proteins released from the cells during the decellularization process. We, therefore, focused our analysis on the previously published set of human core matrisome proteins [31]. From 274 core matrisome proteins, we were able to quantify 82 proteins (Supplementary Figure 3). Principal component analysis showed a close relation between the two FAP+ pericyte‐like cell cultures and HBVP, while ECM produced by U87 glioma cells was highly separated from these cell cultures (Figure 4D). In line with ELISA results in cell lysates, the highest abundances of COLI (both collagen alpha‐1(I) chain and collagen alpha‐2(I) chain) and FN1 were measured in samples derived from FAP+ pericyte‐like cells and HBVP. Compared to U87 glioma cells, FAP+ pericyte‐like cells and HBVP had 15–44 times higher abundance of collagen alpha‐1(I) chain, 16–31 times higher abundance of collagen alpha‐2(I) chain proteins, and 21–68 times higher abundance of FN1 (Figure 4E). FN1 was detectable in U87‐derived samples in small quantities. Interestingly, only one core matrisome protein, decorin, had significantly higher abundance in U87 samples compared to FAP+ pericyte‐like cell cultures. Further analysis revealed that 20 out of the 82 quantifiable core matrisome proteins were essential constituents of basement membranes including collagen IV, laminins, and nidogens. Most of these proteins were more abundant in ECM samples derived from FAP+ pericyte‐like cell cultures than in U87 (Figure 4F, Supplementary Table 4). Collectively, our findings demonstrate the crucial role of FAP+ pericyte‐like cells in the expression and deposition of COLI and FN1 within the GBM microenvironment. Moreover, they suggest the involvement of the FAP+ pericyte‐like cells in the secretion of essential basement membrane proteins, which together with COLI and FN1 may create a permissive environment for glioma cell dissemination.

### 2.4 ECM produced by FAP+ pericyte‐like cells enhances glioma cell migration, adhesion, and FAK activation

**FIGURE 5:**
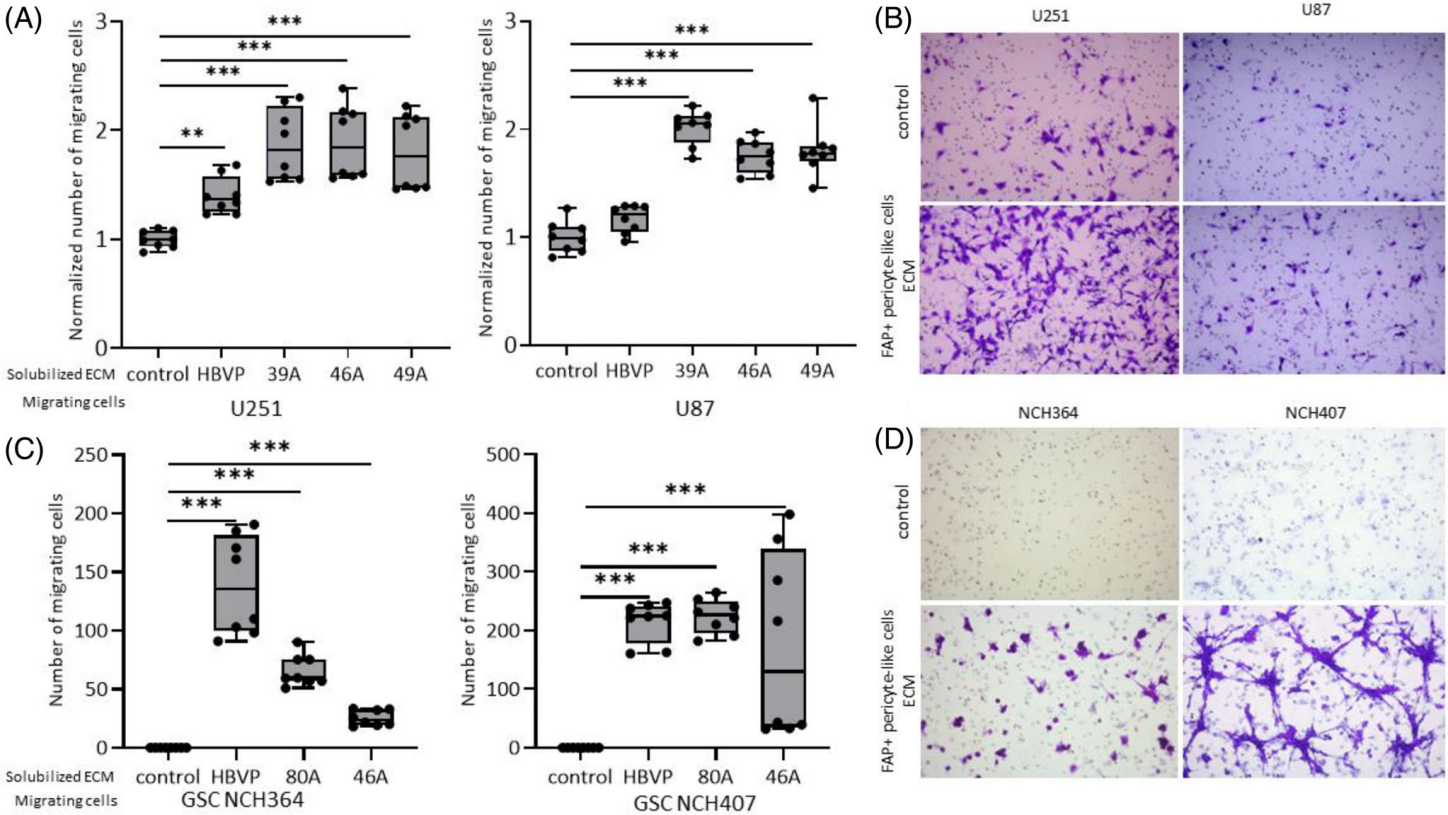
Extracellular matrix produced by FAP+ pericyte‐like cells enhances glioma cell migration. (A) Haptotaxis of U251 and U87 cells through inserts coated with ECM produced by FAP+ pericyte‐like cells (39A, 46A, 49A) and HBVP, respectively. Data in each experiment were normalized to the migration of glioma cells on uncoated inserts. (B) Representative images of migrating U251 and U87 glioma cells. (C) Haptotaxis of glioma stem‐like cells NCH364 and NCH407 through inserts coated with ECM produced by FAP+ pericyte‐like cells (80A, 46A) and HBVP. Results are presented as a number of migrating cells per visual field (10× objective). (D) Representative images of migrating NCH364 and NCH407 glioma stem‐like cells. Box—10th to 90th percentile, line—mean, whiskers—min‐max values, dots—raw data. Results of two independent experiments performed in quadruplicates, **p <0.01, ***p <0.001 compared to the corresponding control, Kruskal–Wallis test.

**FIGURE 6:**
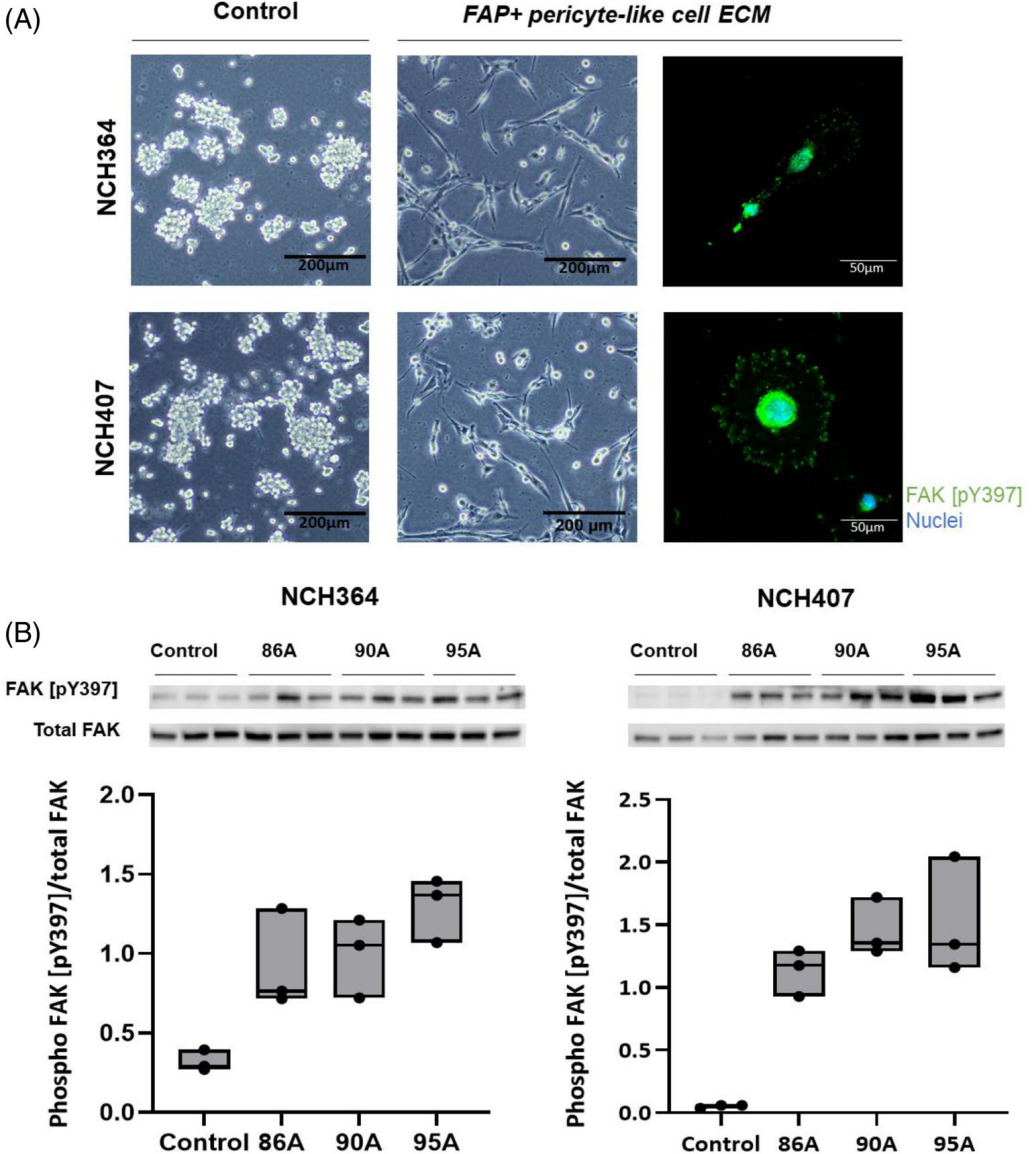
Extracellular matrix produced by FAP+ pericyte‐like cells induces glioma cell adhesion and activates FAK signaling. (A) Representative phase contrast images and immunodetection of phospho FAK [pY397] in glioma stem‐like cells NCH364 and NCH407 seeded on an uncoated surface (control) or on a surface coated with ECM produced by FAP+ pericyte‐like cells. (B) Western blot detection of phospho FAK [pY397] and total FAK in NCH364 and NCH407 18 h after seeding on an uncoated surface (control) or on a surface coated with ECM produced by FAP+ pericyte‐like cells (86A, 90A, 95A). Ratio between total protein (stain‐free method)‐normalized phospho FAK [pY397] and total FAK was used to assess the activation of FAK signaling. The experiment was performed in three biological replicates. Box—min and max, line—median, dots—raw data.

To assess the effect of ECM produced by FAP+ pericyte‐like cells on glioma cell migration, we coated the bottom side of transwell inserts with solubilized ECM and performed a haptotaxis assay. ECM produced by U87 glioma cells had no effect on the migration of U251 cells (Supplementary Figure 4). In contrast, all three ECMs produced by FAP+ pericyte‐like cells significantly increased the migration of U251 and U87 cells (1.8–2.1‐fold and 1.7–2.0‐fold increase, respectively) compared to uncoated inserts (Figure 5A, B). ECM produced by HBVP had a more limited effect and only increased the migration of U251 cells (1.42‐fold increase). To confirm these results, the experiments were repeated using patient‐derived GSC cultures. Migrating cells were observed only in inserts coated with ECM produced by FAP+ pericyte‐like cells and HBVP. Both GSC cultures were unable to migrate through uncoated inserts (Figure 5C, D), probably because of their low ability to adhere to the uncoated surfaces. Accordingly, GSCs efficiently adhered and formed plasma membrane protrusions containing phosphorylated FAK only on ECM‐coated surfaces (Figure 6A). Higher FAK activation in cells adhering to ECM produced by FAP+ pericyte‐like cells compared to cells adhering to uncoated plastic was confirmed by WB analysis (Figure 6B).

## 3 DISCUSSION

In this report, we show that COLI and FN1 are more abundantly expressed in GBM compared to non‐malignant brain tissue. Moreover, to the best of our knowledge, we are the first to establish FAP+ pericyte‐like cells as crucial producers of a complex ECM rich in collagen I and fibronectin in GBM. In vitro, FAP+ pericyte‐like cells deposit the fibrillary proteins in the form of a 3D ECM and produce significantly higher levels of COLI and FN1 compared to other cell types residing in the GBM microenvironment. In addition to COLI and FN1, the ECM produced by FAP+ pericyte‐like cells is enriched in several key basement membrane proteins. Finally, we demonstrate that this ECM facilitates glioma cell migration and adhesion and activates FAK signaling. Collectively, our results support the conclusion that FAP+ pericyte‐like cells contribute to glioma cell dissemination.

FAP is a characteristic marker of mesenchymal cells, such as cancer‐associated fibroblasts and pericytes, in many types of malignancies [32]. FAP+ mesenchymal cells play an important role in maintaining tumor‐permissive environment in various epithelial cancers [33, 34, 35, 36], among others by depositing COLI and FN1, which promote tumor invasion [36, 37, 38]. The presence and possible importance of FAP+ mesenchymal cells in brain tumors is less explored. Results from single‐cell transcriptomic analyses suggested that FAP+ pericyte‐like cells in GBM may contribute to the production of fibrillar ECM [26]. Recently, we provided evidence that FAP+ cancer‐associated fibroblasts are present in brain metastases of various origins and showed that these cells are localized in COLI‐rich areas [39]. Furthermore, we identified FAP+ pericyte‐like cells in the PVN of GBM and described their role in GBM angiogenesis [10, 27]. This, together with the characteristic expression of FAP during tumorigenesis, makes FAP+ mesenchymal cells a promising candidate target for new theranostic approaches [40, 41].

ECM of the brain is unique compared to other tissues. Essential constituents of the brain ECM include hyaluronic acid, proteoglycans, and glycosaminoglycans. Conversely, typical fibrillar ECM proteins constitute minor components of the brain ECM, concentrated in the PVN in the healthy brain. Gliomagenesis is associated with major alterations in the expression pattern of ECM proteins [14, 42, 43, 44]. The literature evidence regarding COLI expression in GBM is rather scarce and contradictory. Zamecnik et al. did not detect COLI by immunohistochemistry [45]; however, later studies reported expression of COLI and its internalization receptor endo180 in GBM [13] and correlation between higher expression of COL1A2 and poor survival [46, 47]. In addition, CD133‐positive glioma cells have been found in close proximity to COLI in perivascular areas in GBM [11]. Our analysis of transcriptomic data and ELISA results clearly demonstrate that COLI expression is significantly elevated in GBM compared to non‐tumorous brain tissue. We further found a positive association between COLI expression and the presence of FAP, a marker of pericyte‐like cells in GBM. COLI and FAP were primarily found in the perivascular regions and vascular structures with high FAP exhibited higher expression of *COL1A1* and *COL1A2* genes. Pericytes residing in the PVN play a crucial role in blood vessel maturation, among others by the deposition of basement membrane proteins [48]. Under the influence of cancer cells, pericytes upregulate the expression and deposition of ECM [49, 50]. Our results show that FAP+ pericyte‐like cells isolated from human GBMs express high amounts of COLI in vitro. HBVP produced comparable amounts, probably because these cells are derived from fetal tissue. Production of COLI by other cell types was very low. These observations are in concordance with literature data that FAP+ mesenchymal cells, including cancer‐associated fibroblasts, are the main producers of COLI in epithelial tumors [51, 52].

Similar to COLI, we observed a higher expression of FN1 in GBM compared to non‐tumorous brain and its association with FAP expression. The role of FN1 in gliomagenesis has been widely studied and FN1 has been proposed to be a negative prognostic marker in GBM [20]. Consistent with previous reports demonstrating FN1 expression in endothelial cells in colorectal and breast cancer [53], our immunohistochemistry findings revealed that FN1 was primarily expressed in the perivascular regions in GBM. In line with that, FN1 was abundantly expressed in FAP+ pericyte‐like cells and microvascular endothelial cells isolated from GBM in vitro. Our results based on several patient‐derived GSC cultures indicate that FN1 expression in glioma cells is low. This contrasts with a previous study using permanent glioma cell lines [43]. However, it is well established that serum‐cultured glioma cells may undergo mesenchymal drift and typically upregulate expression of various ECM proteins [54].

In vitro‐produced 3D‐extracellular matrices represent a well‐established and suitable tool to investigate the composition of extracellularly deposited proteins. In our study, we performed MS analysis to assess the global composition of the 3D‐extracellular matrices produced by FAP+ pericyte‐like cells. As expected, COLI and FN1 were highly abundant. At low levels, COLI and FN1 was detected also in U87‐produced ECM. This could be explained by modest expression of both proteins in these cells as shown by us and others [13, 55]. FN1 was further identified in control, gelatin‐coated plastic. This might be caused by the presence of bovine fibronectin in culture medium. Bovine FN1 has high sequence homology to human FN1 and was therefore identified in our analysis. Collectively, these results suggest that FAP+ pericyte‐like cells abundantly produce and deposit fibrillar ECM proteins, whereas glioma cells can express fibrillar ECM proteins [43], but their ability to deposit these proteins in the extracellular space is strongly limited.

In addition to COLI and FN1, several other ECM proteins were more abundant in the 3D extracellular matrices produced by FAP+ pericyte‐like cells compared to glioma cells. The majority of these are proteins constituting basement membranes and promoting cell adhesion. Overexpression and deposition of fibrillar ECM in PVN seems to have crucial impact on the invasion capacity of glioma cells [42]. Previous studies using synthetic hydrogels have demonstrated that the protein composition, concentration, and resulting stiffness of the ECM affect the invasiveness of glioma cells [17, 42, 46]. A possible limitation of these studies is that they mostly used individual ECM proteins. The complex ECM produced by FAP+ pericyte‐like cells increased the migration of various glioma cell lines including GSC cultures. These findings build upon our previous observation that FAP+ pericyte‐like cells promote glioma cell migration via soluble factors [27], providing additional insights into the possible mechanisms by which FAP+ pericyte‐like cells may contribute to GBM progression.

GSC cultures represent state‐of‐the‐art models for GBM research and recapitulate the phenotype of glioma cells better than established cell lines propagated in serum‐containing media [56]. One of the possible mechanisms that might promote GSC adhesion and migration is the ECM‐mediated activation of FAK. GSC were unable to adhere and migrate in the absence of ECM produced by FAP+ pericyte‐like cells. We observed a strong activation of FAK in both GSC cultures when in contact with ECM produced by FAP+ pericyte‐like cells. FAK is a protein kinase activated by integrins, which subsequently leads to increased cell adhesion and migration [24]. It has been previously described that COLI and FN1 signaling through integrins promotes the expression of stem cell markers such as CD133, SOX2, and nestin and enhances glioma cell aggressiveness [11, 46, 47, 57]. Thus, FAK signaling may be involved in adhesion and migration of GSC and regulate their stemness phenotype.

Our study has certain limitations that need to be acknowledged. In our analyses of publicly available datasets and our ELISA studies, we used the expression of FAP as a proxy to estimate the quantity of FAP+ mesenchymal cells. However, it is important to note that FAP can also be expressed in cancer cells in GBM, as reported by us and others [10, 26, 58]. Nevertheless, the potential inaccuracy introduced by this approach is relatively minor‐less than 30% of GBMs contain FAP+ cancer cells, which in most cases constitute less than 10% of the cells [27]. Furthermore, it should be recognized that the in vitro assay used to analyze the spectrum of ECM proteins deposited in the extracellular space by individual cell types may not fully replicate the complex microenvironmental cues experienced by the cells in vivo. In the case of glioma cells, in vitro culture has been shown to induce the expression of various collagens and laminin [54]. Despite that, we found that the expression and deposition of most ECM proteins in FAP+ mesenchymal pericyte‐like cells were higher, and correlative immunohistochemical analyses in GBM tissues supported the conclusions drawn from our in vitro studies.

## 4 CONCLUSION

Collectively, we provide a new insight into the cellular source of fibrillar ECM proteins in GBM and how these proteins promote the malignant phenotype of glioma cells. We identify FAP+ pericyte‐like cells as the main producers of ECM rich in COLI and FN1 in GBM and provide evidence that this ECM facilitates migration and adhesion of glioma cells through FAK activation.

## 5 MATERIALS AND METHODS

### 5.1 Patient samples

Tissue samples were obtained from surgically treated patients with GBM (WHO grade IV glioma, IDHwt, *n* = 52) and PRE (*n* = 10; controls) at the Military University Hospital and the Na Homolce Hospital and were stored at −80°C. The study was approved by the Institutional Ethics Committee (study approval numbers 108‐39/4‐2014‐UVN and 7/8/2014‐25) and conducted in accordance with the Declaration of Helsinki. Full informed consent was obtained from all donors prior to neurosurgical resection. GBM diagnosis was established according to the current WHO classification [1], and patients received no preoperative oncological treatment. FAP high (upper tercile) and FAP low (lower tercile) GBM tissues were selected from our tissue biobank based on FAP protein expression, which was determined in a previous study [29].

### 5.2 Cell lines and cell culture

Cells were cultured under standard conditions at 37°C in a humidified atmosphere of 5% CO_2_ in air. Glioma cell lines U251 and U87 were obtained from ATCC (LGC Standards, Teddington, UK) and cultured in Dulbecco's Modified Eagle's Medium (DMEM, Merck, Darmstadt, Germany) supplemented with 10% fetal calf serum (FCS, Merck). HBVP were acquired from ScienCell (ScienCell Research Laboratories, Carlsbad, CA, USA) and cultured under conditions recommended by the provider in pericyte medium (PM) supplemented with 2% fetal bovine serum (FBS), pericyte growth supplement (PGS), 100 units/mL penicillin G, and 100 μg/mL streptomycin (P/S, all provided by ScienCell Research Laboratories). The phenotype of the cells was regularly validated using immunocytochemistry (Supplementary Figure 5). GSCs were derived from freshly resected GBM tissues as previously described [59]. Briefly, tumor tissue was dissociated using a Papain Dissociation System (Worthington Biochemical Corporation, USA). Tissue samples were cleared of macroscopic vessels and necrotic tissue and finely minced using a sterile scalpel. The tissue pieces were incubated with papain solution and dissociated by pipetting. After incubation, papain was quenched by albumin‐ovomucoid solution and the resulting single‐cell suspension was cultured in DMEM/F‐12 (Sigma‐Aldrich Aldrich, Czech Republic), 1% GlutaMAX, 2% B‐27™ Supplement minus vitamin A (Gibco, Thermo Fisher Scientific, USA), 100 U/mL penicillin, 100 μg/mL streptomycin (Sigma‐Aldrich, Czech Republic), 20 ng/mL EGF and 20 ng/mL FGF (PeproTech EC Ltd., London, UK) in non‐adherent cell culture flasks. Glioma‐associated endothelial cells were derived from freshly resected GBM tissue as previously described [27]. Briefly, mechanically and enzymatically digested tissue pieces were cultured on fibronectin‐coated (3 μg/cm^2^) flasks in an initiation medium (RPMI‐1640 supplemented with 10% FCS, 10% Nu serum (BD Bioscience, Franklin Lakes, NJ, USA)), 1 mM HEPES (Merck), 300 UI Heparin (Zentiva, Prague, Czech Republic), 1% P/S (Sigma‐Aldrich), and 3 μg/mL endothelial cell growth supplement (ECGS, BD Bioscience). Cells migrating from the explants were harvested and processed by indirect magnetic‐activated cell sorting (MACS) using an anti‐CD105 antibody (M3527, Dako Agilent, Santa Clara, CA, USA, 0.15 μg per 1 × 106 cells at 4°C for 8 min). After 2 weeks, 10% Nu Serum was removed from the culture media, and ECGS concentration was increased to 30 μg/mL. Macrophages were differentiated from monocytes obtained from buffy coats of healthy donors. Buffy coats (*n* = 2) were collected at the Institute of hematology and blood transfusion, Prague (ethical committee approval reference number 06/11/2020); informed consent was obtained from all subjects. Peripheral blood mononuclear cells (PBMC) were isolated by density gradient centrifugation. The buffy coat sample was layered on Ficoll Paque (Cytiva) and centrifuged (500 g, 40 min, RT). The PBMC fraction was isolated using a Pasteur pipette, washed twice with phosphate‐buffered saline (PBS), and red blood cells were lysed using RBC lysis solution (Miltenyi Biotec, Bergisch Gladbach, Germany). Monocytes were isolated from PBMC using negative selection MACS (pan‐monocyte isolation kit, Miltenyi). The purity of the isolated monocytes, determined as a percentage of CD14 positive cells, was measured by flow cytometry (CD14‐FITC, Sony, San Jose, CA, USA) and was >90%. Monocyte‐derived macrophages were obtained after 7 days of culturing in RPMI‐1640 medium supplemented with 10% heat‐inactivated FCS and 50 ng/mL M‐CSF (Miltenyi). Fresh culture media were replaced on day four. IL‐4 (20 ng/mL; Miltenyi) was added 24 h prior to harvesting. The M2‐like phenotype was validated by the expression of CD163 and CD206 and the absence of CD80 (CD163‐PE; CD206‐APC; CD80‐APC; Sony). Cancer cell lines were used for up to 8 passages, and FAP+ pericyte‐like cells and glioma‐associated endothelial cells were used for up to five passages after derivation. Mycoplasma contamination was ruled out using a PCR‐based assay.

### 5.3 Derivation and culture of FAP+ pericyte‐like cells

FAP+ mesenchymal cells were derived from human GBM tissue as previously described [27]. Briefly, freshly resected human GBM tissue was cut into small pieces, digested at 37°C for 20 min with TrypLE Select (Thermo Fisher Scientific), placed in fibronectin‐coated (3 μg/cm^2^, Merck) cell culture flasks, and cultured in PM supplemented with 2% FBS, PGS, and P/S (ScienCell Research Laboratories) for 7 days. Cells migrating from explants were harvested and used for indirect MACS according to the manufacturer's instructions with minor modifications. Cells were resuspended at 1 × 10^7^ cells/mL and incubated with 0.1 μg of anti‐FAP F11‐24 antibody (Santa Cruz Biotechnology, Dallas, TX, USA) per 1 × 10^6^ cells (4°C, 8 min). Labeled cells were resuspended at 1 × 10^7^ cells/mL, then 0.15 μL of magnetic DynaBeads (Thermo Fisher Scientific) were added per 1 × 10^6^ cells, and the resulting suspension was incubated at 4°C for 4 min. The FAP+ fraction was harvested and cultured in fibronectin‐coated flasks in PM supplemented with 2% FBS, PGS, and P/S.

### 5.4 Preparation of cell‐derived 3D extracellular matrices

Deposition of ECM was evaluated using a 3D ECM assay as previously described [30] with minor modifications. Briefly, 35 mm plates were precoated with 0.2% gelatin (1 h, 37°C, gelatin from cold water fish, Sigma‐Aldrich). The plates were washed with PBS and 2 mL of filtered 1% glutaraldehyde in PBS was added to crosslink gelatin (30 min, RT). The glutaraldehyde solution was discarded, and the plates were washed three times with PBS. To remove the remaining glutaraldehyde, 2 mL of 1 M ethanolamine in sterile water was added and incubated (30 min, RT). Ethanolamine was discarded and plates were washed three times with PBS. 2 mL of complete culture medium was added and FAP+ pericyte‐like cells, HBVP, or glioma cells were seeded at 5 × 10^5^ cells per plate. Media supplemented with 50 μg/mL ascorbic acid were exchanged every other day for 8 days to obtain 3D matrices. The matrices were decellularized using an alkaline detergent treatment (0.5% Triton X‐100, 20 mM NH_4_OH in PBS for 5 min at 37°C). For immunohistochemical analyses of the 3D matrices, cells were seeded onto gelatin‐precoated coverslips and processed identically.

### 5.5 Immunofluorescence staining, image analysis

Single, double, and triple immunofluorescence labeling was performed in 10 μm frozen tissue sections, cells, and 3D EC matrices as described previously [10, 27]. Briefly, after fixation with 4% paraformaldehyde (10 min, RT), samples were permeabilized with 0.1% Triton X‐100 (5 min, RT) and blocked with 10% FBS and 1% bovine serum albumin in Tris‐buffered saline (60 min, RT). Primary antibodies against FAP (clone D8, MABS1001, Vitatex, Stony Brook, NY, USA, 1:800, 60 min, RT), collagen type I (COLI, ab34710, Abcam, USA, 1:800, 60 min, RT) Fibronectin (FN1, Ab23750, Abcam, USA, 1:200, 60 min, RT), von Willebrand factor (vWf, LS‐C290244, Dako Agilent, 1:200, 60 min, RT) and glial fibrillary acidic protein (GFAP, 11‐255‐M001, Exbio, 1:200, 60 min RT) were visualized using the corresponding secondary antibodies (anti‐rabbit Alexa fluor 488, A‐11008, anti‐mouse Alexa fluor 546, A‐21202, anti‐rat Alexa fluor 488 A‐21208 and anti‐mouse Alexa fluor 633, A21052, ThermoFisher Scientific, 1:500, 60 min, RT) and 50 ng/mL Hoechst 33258 (Sigma‐Aldrich, St. Louis, MO, USA) was used for nuclear counterstaining. Samples were mounted in Aqua Poly/Mount (Polysciences, Hirschberg, Germany) and viewed and photographed using an IX70 fluorescence microscope (Olympus, Japan) with Orca‐flash 4.0 camera (Hamamatsu, Hamamatsu, Japan). To quantify COLI and FN1 in GBM tissues, five representative fields were photographed for each antigen (COLI and FN1) in each tissue section. In total, 22 different GBM tissue sections were used. The percentage of immunopositive area of COLI and FN1 was determined using the analyze particle plugin in ImageJ (National Institutes of Health, Bethesda, MD, USA).

### 5.6 Confocal microscopy

Tissue sections and decellularized cell‐derived 3D matrices were viewed and imaged using the Stellaris 5 Confocal microscope (Leica, USA) equipped with 488, 546, and 634 nm lasers. Images were analyzed and processed using LAS X software (Leica, USA).

### 5.7 Preparation of tissue and cell lysates

Tissue and cell lysates were prepared as previously described [29]. Briefly, tissue samples were homogenized on ice with Ultra‐Turrax homogenizer (T10; IKA, Königswinter, Germany) in a homogenization buffer (2 mM Na_2_HPO_4_, 0.6 mM KH_2_PO_4_ and 22.4 mM NaCl, pH 6.0). The homogenates were mixed (1:1 v/v) with lysis buffer (10 mM Tris–HCl, pH 7.5, containing 1% Triton X‐100, 0.1% SDS, 100 mM NaCl, 1 mM EDTA, 1 mM EGTA, 10% glycerol and a mix of protease and phosphatase inhibitors including 25 μM pepstatin A, 200 μM AEBSF, 50 μM E‐64, 5 mM NaF, and 1 mM Na_3_VO_4_), mixed using vertical rotator (1 h, 4°C) and centrifuged (22,000 g, 30 min, 4°C). The supernatant was transferred to new microcentrifuge tubes and stored at −80°C. To assess the expression of COLI and FN in vitro, cells were cultured under standard conditions in their corresponding culture media supplemented with ascorbic acid (Sigma‐Aldrich, 50 μg/mL) for 5 days. Cell lysates were prepared from adherently growing cells. Cells were washed three times with ice‐cold PBS and scraped in lysis buffer (9 μL/cm^2^) using a plastic policeman. The lysates were cleared by centrifugation (22,000 g, 30 min, 4°C) and the supernatant was stored at −80°C.

### 5.8 Protein quantification

Protein quantity in tissue and cell lysates was analyzed using a detergent‐compatible protein assay (Biorad Laboratories, Hercules, CA, USA) according to the manufacturer's instructions. The protein concertation of the solubilized cell‐derived 3D matrices was measured by Pierce™ BCA Protein Assay Kit (Thermo‐scientific) according to the manufacturer's instructions. The absorbance was measured using a 96‐well plate reader (Sunrise; Tecan) at 750 nm and 562 nm, respectively.

### 5.9 ELISA

COLI and FN were analyzed in tissue and cell lysates using DuoSet ELISA kits (DuoSet Human collagen I, cat. No. DY6220‐05 and DuoSet Human Fibronectin, cat. No. DY1918‐05 R&D Systems, USA), according to the manufacturer's recommendations.

### 5.10 Western blot analysis

Western blot analysis was performed as described previously [39] with the modifications specified below. Cells were lysed on ice in RIPA lysis buffer (R0278; Sigma‐Aldrich, Germany) with protease and phosphatase inhibitors 18 h after seeding. Immunodetection was performed using the Phospho‐FAK (Tyr397) Polyclonal Antibody (44‐624G; Thermo Fisher Scientific, 1:500 in EveryBlot Blocking Buffer; [EBBB] Bio‐Rad, USA, 4°C overnight), the FAK (C‐20) antibody (sc‐558; Santa Cruz Biotechnology, USA, 1:1000 in 5% milk, 4°C overnight), the HRP‐conjugated secondary antibody (NA934; Amersham, 1:10,000 in EBBB and 5% milk, respectively, 60 min at RT) and Immobilon ECL Ultra Western HRP Substrate (WBULS0100; Millipore). For relative quantification, the phospho FAK [pY397] and total FAK signal were normalized to the total protein signal determined by the stain‐free methodology using ImageLab Software (Bio‐Rad, USA). The ratio between total protein‐normalized activated phospho FAK [pY397] and total FAK was used to evaluate activation of the FAK signaling. The experiment was performed in three biological replicates.

### 5.11 Sample preparation for mass spectrometry

Decellularized matrices and gelatin‐coated petri dishes were harvested using lysis buffer (1% SDS in 50 mM Tris HCl, pH = 7.5, Sigma‐Aldrich). Briefly, 200 μL of preheated (95°C) lysis buffer was added onto the dishes and incubated for 3 min. The material was collected using a plastic policeman, transferred into a microtube, vortexed (30 s, RT), and cleared by centrifugation (20,000 g, 15 min, 20°C). The supernatant was transferred to a new microcentrifuge tube and stored at −80°C. ECM protein lysates were thawed and sonicated in an ultrasonic bath (10 min, 40 kHz, 22°C) and each sample was mixed to the total volume 200 μL with the washing buffer (8 M urea and 5 mM EDTA in 50 mM ammonium bicarbonate—AmBic). Unless stated otherwise, all steps were performed at (RT, 21°C). Microcon 30 K centrifugal ultrafiltration units (Merck Millipore Ltd.) were washed with 50 mM Tris HCl (centrifugation 5 min, 10,000 g), and ECM samples were loaded and centrifuged (15 min, 10,000 g). Eluates were discarded and ultrafiltration units were washed with 200 μL of the washing buffer three more times. Proteins were reduced by 150 μL of 10 mM tris(2‐carboxyethyl) phosphine (TCEP) in a washing buffer (30 min, 32°C) and units were centrifuged. Then, the proteins were alkylated by addition of 150 μL of 40 mM iodoacetamide (IAA) in the washing buffer (35 min, 25°C, in the dark) and centrifuged. Eluates were discarded and units were washed with 150 μL of the washing buffer and centrifuged. Units were then washed three times with 200 μL of 50 mM AmBic and eluates were discarded. Proteins were digested on filter by adding 150 μL of 50 mM AmBic with 0.02% (w/v) ProteaseMAX (Promega) with 1 μg of LysC protease (Wako) at 37°C for 2 h. Then, 1 μg of trypsin (Promega) was added, and samples were incubated at 37°C overnight. Peptides were collected by centrifugation (10,000 g, 10 min). Elution was repeated with 150 μL of 50 mM AmBic (centrifugation 12,000 g, 15 min) and eluates were combined. Peptide samples were acidified by formic acid (FA) to a final concentration of 2% (v/v), centrifuged (20,000 g, 20 min, 4°C) and supernatants were collected. Peptides were then desalted by C18 MicroSpin columns (The Nest Group) according to the manufacturer's instructions. Desalted peptides were dried in vacuum centrifuge at 45°C and stored at 4°C until resuspension in a loading buffer (2% acetonitrile in 0.5% FA). Peptide concentrations were determined at λ = 280 nm (Synergy HTX, BioTek). Retention time normalization peptides (iRT, Biognosys) were added at iRT: sample ratio 1:20 (v/v) to all samples.

### 5.12 Mass spectrometry analyses

The liquid chromatography tandem mass spectrometry (LC–MS/MS) setup was modified from [60]. One μg of each peptide sample was separated in a trap‐elute mode, using the Eksigent nano‐LC 425 (Sciex) online connected to 5600+ TripleTOF (Sciex). Peptides were loaded on Acclaim PepMap 100 C18 trap column (5 μm, 0.1 × 20 mm; Thermo Fisher Scientific) for 10 min at 2 μL/min and then separated by fused‐silica column (25 cm length, 75 μm inner diameter) packed in‐house with ProntoSIL 120 Å 3 μm C18 AQ beads (Bischoff Analysentechnik GmbH). Separation was performed by 130 min linear gradient of acetonitrile (I) with a flow rate of 200 nL/min (5%–35% I in 0.1% FA over 120 min followed by 35%–50% I in 0.1% FA over 10 min). In case of data‐dependent acquisition (DDA) approach, 40 positively charged precursors with the highest intensity in MS1 (mass range 400–1250 Da, accumulation time 300 ms) were fragmented and analyzed in MS2 within high sensitivity mode (mass range 170–2000 Da, accumulation time 100 ms). The cycle time was 4.35 s, precursor exclusion time was 13 s. For quantification analysis, the Sequential Window Acquisition of All Theoretical Mass Spectra (SWATH‐MS) approach with 30 variable windows was used. Variable windows were calculated by a SWATH Variable window calculator (Sciex) based on data from DDA measurement of ECM sample. With respect to DDA settings, SWATH‐MS was acquired in the same mass range. The accumulation time was 150 and 100 ms for MS1 and MS2, respectively, and cycle time was 3.2 s.

### 5.13 Analysis of mass spectrometry data

Identification and quantification of proteins in MS data were performed as we described previously [60, 61]. Briefly, to prepare data for sample‐specific spectral library, DDA data were analyzed in Mascot Distiller 2.7.1 and Mascot Server 2.6.2 (both Matrix Science Ltd.). Human reference proteome (UP000005640) from UniProt [62] (20,607 proteins, one protein sequence per gene, downloaded November 13, 2022) was appended with common protein contaminants and used for protein identification. Mass tolerances in Mascot search were set to 15 ppm and 20 ppm for MS1 and MS2, respectively. Protein modifications were set to carbamidomethylation of cysteine (fixed), and protein N‐terminal acetylation and oxidation of methionine (both variable). Enzyme specificity was set to always cleavage after lysine, and cleavage after arginine, unless proline followed, with two missed cleavages allowed. The SWATH‐MS data were analyzed in Skyline‐daily (version 22.2.1.351) [63] using sample‐specific spectral library created in Skyline from 16 DDA measurements searched by Mascot (Mascot expectation score threshold 0.01). MS2 filtering of data was performed with resolving power 25,000 in high‐selectivity extraction mode. The iRT peptides were used to align retention times between samples and retention time filtering was set to select only peaks within ±5 min of predicted retention time. Duplicated and repeated peptides were removed and only proteins with at least two peptides (minimally three transitions per peptide) were further processed. The mProphet [64] was trained on shuffle sequence method generated decoy peptides and then used for automatic peak picking. The quantitative information for each transition (transition intensity defined as an area under the curve, information about retention time, and detection q‐value) was exported from Skyline and further processed in R (version 4.2.3) [65] using Msstats R package (version 4.6.5) [66] to obtain individual protein abundances. The iRT peptides, peptides with oxidized methionine, and peptides with *q* >0.01 were removed from the data using SkylinetoMSstats function. For between‐run normalization, all intensity values were scaled by a factor calculated as global median total ion current (TIC) divided by run‐specific median TIC. Protein abundances for each run were calculated from log2‐transformed transition intensities using Msstats dataProcess function, with Tukey median polish set as a summary method, followed by quantification (type = Sample).

### 5.14 Solubilization of cell‐derived 3D extracellular matrix for in vitro assays

Decellularized matrices were solubilized on ice using a solubilization buffer (5 M guanidine hydrochloride (Sigma‐Aldrich) and 10 mM dithiothreitol (Sigma‐Aldrich) dissolved in sterile dH_2_O) [67]. Briefly, plates with decellularized matrices were washed with ice‐cold PBS, any excess PBS was then completely removed. Afterwards, 300 μL of solubilization buffer was added. After 5 min, the matrices were scraped using a plastic policeman and transferred to a microcentrifuge tube. The solution was vortexed and placed on a vertical rotator (1 h, 4°C). Subsequently, the solution was centrifuged (12,000 g for 15 min at 4°C), and the supernatant was transferred into a new microcentrifuge tube and stored at 4°C for a maximum of 1 week.

### 5.15 Haptotactic transwell migration assay, adhesion of GSCs

Solubilized matrices were diluted 1:12.5 in a solubilization buffer (described above). A total of 60 μL drops were applied at the bottom of a 100 mm plate and individual inserts (6.5 mm diameter, 8 μm pores, Corning, USA) were placed into these drops and incubated (1 h, RT). The inserts were subsequently washed three times with PBS and transferred into 24‐well plates containing serum‐free media (DMEM resp. DMEM F12 with GlutaMAX (1%)), B‐27™ Supplement minus vitamin A (2%), 100 U/mL penicillin, 100 μg/mL streptomycin. Glioma cells (6 × 10^4^) were seeded into the transwell inserts and allowed to migrate for 24 h. Non‐migrated cells were removed using a cotton swab; transmigrated cells on the lower surface of the insert were fixed with 10% neutral buffered formalin and stained with crystal violet (20 min, RT, Sigma‐Aldrich). Inserts were washed in water and allowed to dry out. Five microscopic fields per insert were photographed (20× objective), and cells were counted manually. Cell adhesion was visually evaluated in GSCs seeded on uncoated dishes and dishes coated with ECM produced by FAP+ pericyte‐like (1:12.5 in a solubilization buffer) 18 h after seeding.

### 5.16 Analysis of the cancer genome atlas (TCGA) database data and IVY glioblastoma dataset

Expression data of primary, IDHwt glioblastomas (*n* = 357) and non‐tumorous brain tissues (*n* = 10) from the TCGA GBM (HG‐UG133A platform) were downloaded from the GlioVis data portal http://gliovis.bioinfo.cnio.es/ on December 17, 2021. Expression of *COL1A1*, *COL1A2*, and *FN1* in distinct regions of GBM were determined using the Ivy Glioblastoma Project data [28], which used laser microdissection followed by RNA sequencing of various micro‐anatomical regions in 10 GBMs. Gene‐level fragments per kilobase per million (FPKM) values were downloaded from the Ivy GAP data portal on October 29, 2021.

### 5.17 Statistical analyses

Statistical analyses were performed with GraphPad Prism 8.0.2 (GraphPad Software, San Diego, CA, USA). Unpaired *t*‐test or ANOVA for normally distributed data and Mann–Whitney U‐test or Kruskal–Wallis test were used to verify the difference between groups. A two‐sided *p* <0.05 was considered statistically significant. PCA biplot and heatmaps of protein abundances from LC–MS/MS experiments were created in R (version 4.2.3) [65]. Briefly, missing values in log_2_ transformed matrix of protein abundances were replaced by 0 if missing completely from at least 1 condition or imputed using knn approach in case of 1 or 2 missing values out of 4 replicates per given condition, using DEP (version 1.2.0) package [68]. PCA biplot of first two principal components was displayed using DEP plot_pca command. Heatmaps were then created using the same data matrix and ComplexHeatmap (version 2.14.0) package [69], with complete hierarchical clustering of rows and columns using Euclidean distance method.

## AUTHORS CONTRIBUTIONS

**Petr Vymola:** Conceptualization, formal analysis, funding acquisition, investigation methodology, project administration, visualization, writing – original draft preparation, writing – review & Editing. **Elena Garcia Borja:** Investigation, visualization, writing – original draft preparation. **Jakub Cervenka:** Data Curation, investigation, writing – original draft preparation. **Barbora Vymolova:** Resources, methodology, writing – original draft preparation. **Eva Balaziova:** Investigation, resources. **Jana Veprkova:** Investigation. **Petr Vodicka:** Formal Analysis, methodology, visualization. **Helena Skalnikova:** Resources. **Robert Tomas:** Resources. **David Netuka:** Resources. **Petr Busek:** Conceptualization, formal analysis, supervision, validation, writing – review & editing. **Aleksi Sedo:** Conceptualization, funding acquisition, supervision, writing – review & editing.

## FUNDING INFORMATION

This work was supported by the Ministry of Education, Youth and Sports of the Czech Republic projects National Institute for Cancer Research (Programme EXCELES, ID Project No. LX22NPO5102) funded by the European Union—Next Generation EU, EATRIS‐CZ (LM2023053), the project Center for Tumor ecology (reg. n. CZ.02.1.01/0.0/0.0/16_019/0000785) supported by the Operational Program Research, Development and Education, Ministry of Health of the Czech Republic (grant NV19‐03‐00501), and Charles University Cooperatio Program, research area” “Oncology and Haematology” and project GA UK no. 1084120.

## CONFLICT OF INTEREST STATEMENT

The authors declare no competing or financial interests.

## Supporting information


**Supplementary Figure 1:** COLI genes and FN1 are expressed at higher levels in GBM compared to non‐tumorous brain tissue. TCGA database‐based bioinformatic analysis of COL1A1, COL1A2, and FN1 expression in GBMs (*n* = 357) and non‐tumorous brain tissue (*n* = 10). ***p < 0.001, Mann–Whitney test.
**Supplementary Figure 2:** Glioma cells do not express COLI and FN1. A representative image of collagen I (COLI) and FN1 expression; expression of glial fibrillary acidic protein (GFAP) was used to identify glioma cells.
**Supplementary Figure 3:** Mass spectrometry analysis of cell‐derived 3D matrices produced by FAP+ pericyte‐like cultures, HBVP, and U87 glioma cell. Relative protein abundance of 82 identified matrisome proteins produced by FAP+ pericyte‐like cells (46A, 80A), HBVP, and U87 glioma cells. Columns represent cell lines (in quadruplicates) and rows represent expressed proteins. Color in each tile represents the scaled log2 abundance value.
**Supplementary Figure 4:** Extracellular matrix produced by U87 glioma cells does not facilitate glioma cell migration. (A) Haptotaxis of U251 through inserts coated with ECM produced by U87 glioma cells. Data in each experiment were normalized to migration of glioma cells on uncoated inserts. Results from two independent experiments performed in quadruplicates, Box—10th to 90th percentile, whiskers—min‐max values, dot—raw data, line—mean, *p* >0.05, Mann–Whitney U test.
**Supplementary Figure 5:** Validation of pericyte characteristics of human brain vascular pericytes (HBVP). HBVP expressed Neural/glial antigen 2 (NG2), platelet‐derived growth factor receptor beta (PDGFRβ), TE‐7, α‐smooth muscle actin (α‐SMA). Expression of sex‐determining region Y (SOX2), Glial fibrillary acidic protein‐(GFAP) was negative. Expression was determined by immunucytochemistry, representative images are shown.
**Supplementary Table 1: P**rotein yields from cell‐derived 3D matrices produced by various cell types. Numbers are means from four biological replicates ± standard deviations. Protein concentrations in U251 and gelatin samples were under the limit of detection.

**Supplementary Table 2.** Proteins detected using the SWATH‐MS approach in gelatin‐coated plates.

**Supplementary Table 3.** Proteins detected using the SWATH‐MS approach in decellularized extracellular matrices produced by 46A and 80A FAP+ pericyte‐like cells isolated from human glioblastomas, human brain vascular pericytes (HBVP), and U87 glioma cells. NA = not detectable. Numbers represent log2 abundance values.

**Supplementary Table 4.** Statistical analysis of 82 matrisome protein abundances detected using the SWATH‐MS approach in decellularized extracellular matrices produced by 46A and 80A FAP+ pericyte‐like cells isolated from human glioblastomas, human brain vascular pericytes (HBVP), and U87 glioma cells. Label (comparison), Log2FC (log2 fold change), SE (log2FC standard error), *T*‐value (test statistic of the Student test), DF (degree of freedom of the Student test), *p*‐value (raw *p*‐value), adj. *p*‐value (*p*‐values adjusted using the approach by Benjamini and Hochberg 1955), issue (issue column shows if there is any issue for inference in corresponding protein and comparison), Missing Percentage (number of measured intensities/ total number of intensities by protein, total number of intensities = number of features * the number of runs in a protein). Imputation Percentage (number of imputed intensities/total number of intensities by protein).

## Data Availability

The dataset from all SWATH‐MS measurements for this study can be found in the Panorama Public repository (https://panoramaweb.org/ECMinGBM.url) and on ProteomeXchange under ID PXD045057. All data uploaded into Panorama Public repository are in private mode before manuscript publication.
