# Supplementary material for: Fibrillar extracellular matrix produced by pericyte‐like cells facilitates glioma cell dissemination
Source: Brain Pathol. 2024 May 5;34(6):e13265. doi: 10.1111/bpa.13265 (PMC11483521; doi:10.1111/bpa.13265)
Supplement: Supplementary file 1 — Supplementary Figure 1: COLI genes and FN1 are expressed at higher levels in GBM compared to non‐tumorous brain tissue. TCGA database‐based bioinformatic analysis of COL1A1, COL1A2, and FN1 expression in GBMs (n = 357) and non‐tumorous brain tissue (n = 10). ***p < 0.001, Mann–Whitney test. Supplementary Figure 2: Glioma cells do not express COLI and FN1. A representative image of collagen I (COLI) and FN1 expression; expression of glial fibrillary acidic protein (GFAP) was used to identify glioma cells. Supplementary Figure 3: Mass spectrometry analysis of cell‐derived 3D matrices produced by FAP+ pericyte‐like cultures, HBVP, and U87 glioma cell. Relative protein abundance of 82 identified matrisome proteins produced by FAP+ pericyte‐like cells (46A, 80A), HBVP, and U87 glioma cells. Columns represent cell lines (in quadruplicates) and rows represent expressed proteins. Color in each tile represents the scaled log2 abundance value. Supplementary Figure 4: Extracellular matrix produced by U87 glioma cells does not facilitate glioma cell migration. (A) Haptotaxis of U251 through inserts coated with ECM produced by U87 glioma cells. Data in each experiment were normalized to migration of glioma cells on uncoated inserts. Results from two independent experiments performed in quadruplicates, Box—10th to 90th percentile, whiskers—min‐max values, dot—raw data, line—mean, p >0.05, Mann–Whitney U test. Supplementary Figure 5: Validation of pericyte characteristics of human brain vascular pericytes (HBVP). HBVP expressed Neural/glial antigen 2 (NG2), platelet‐derived growth factor receptor beta (PDGFRβ), TE‐7, α‐smooth muscle actin (α‐SMA). Expression of sex‐determining region Y (SOX2), Glial fibrillary acidic protein‐(GFAP) was negative. Expression was determined by immunucytochemistry, representative images are shown. Supplementary Table 1: Protein yields from cell‐derived 3D matrices produced by various cell types. Numbers are means from four biological replicates ± stan [file BPA-34-e13265-s003.docx]

**Supplementary material**

***
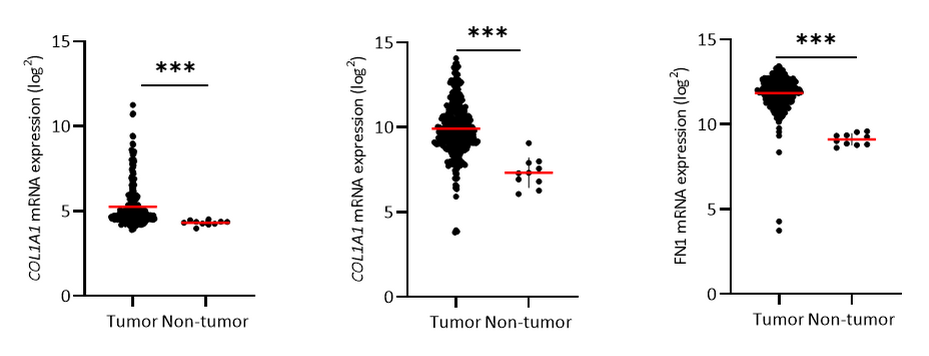
***

***Supplementary fig. 1: COLI genes and FN1 are expressed at higher levels in GBM compared to non-tumorous brain tissue.*** *TCGA database-based bioinformatic analysis of COL1A1, COL1A2 and FN1 expression in GBMs (n=357) and non-tumorous brain tissue (n=10). *** p < 0.001, Mann–Whitney test.*

**
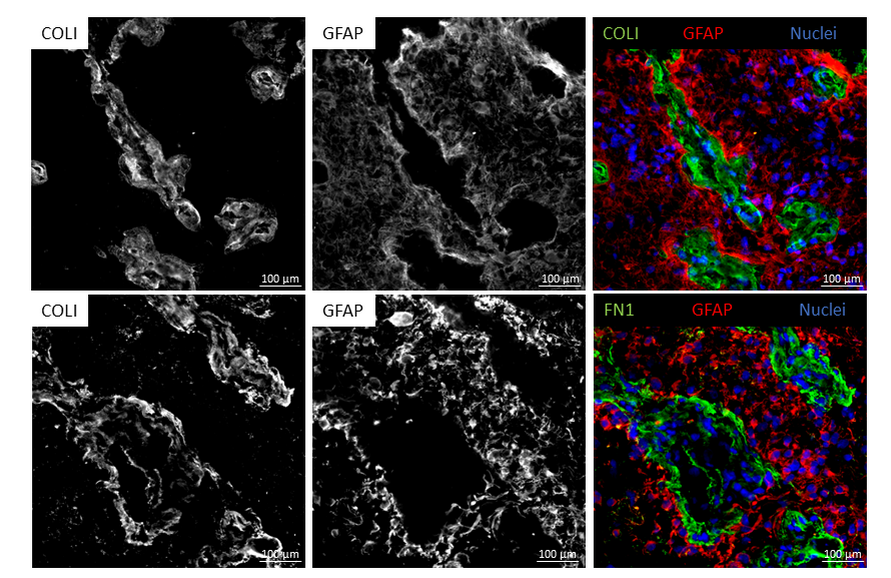
**

**Supplementary fig. 2: Glioma cells do not express COLI and FN1.** *A representative image of collagen I (COLI) and FN1 expression; expression of glial fibrillary acidic protein (GFAP) was used to identify glioma cells.*

***
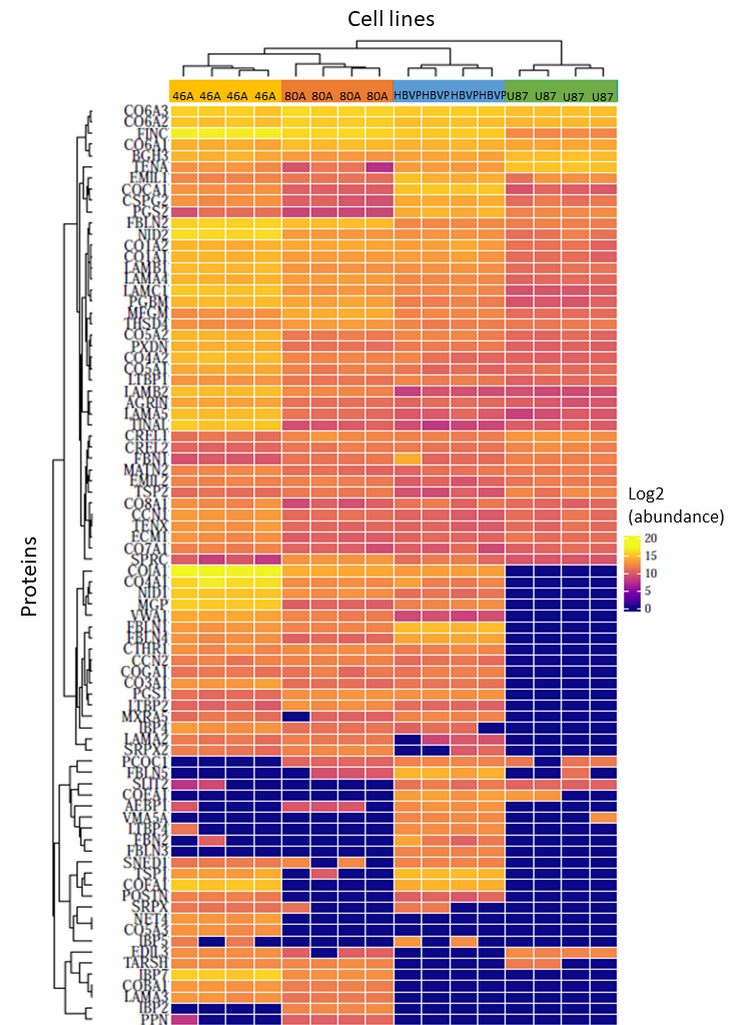
***

***Supplementary fig. 3: Mass spectrometry analysis of cell-derived 3D matrices produced by FAP+ pericyte-like cultures, HBVP and U87 glioma cell.*** *Relative protein abundance of 82 identified matrisome proteins produced by FAP+ pericyte-like cells (46A, 80A), HBVP and U87 glioma cells. Columns represent cell lines (in quadruplicates) and rows represent expressed proteins. Color in each tile represents the scaled log2 abundance value. ​*

*
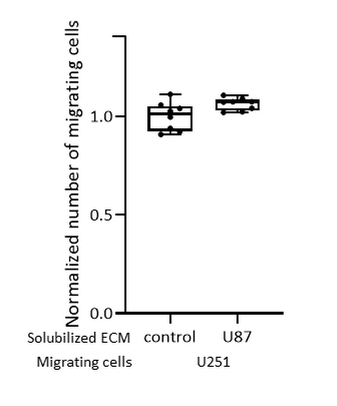
*

***Supplementary fig. 4: Extracellular matrix produced by U87 glioma cells does not facilitate glioma cell migration.*** *A) Haptotaxis of U251 through inserts coated with ECM produced by U87 glioma cells. Data in each experiment were normalized to migration of glioma cells on uncoated inserts. Results from two independent experiments performed in quadruplicates, Box—10^th^ to 90^th^ percentile, whiskers—min-max values, dot—raw data, line—mean, p > 0.05, Mann-Whitney U test.*


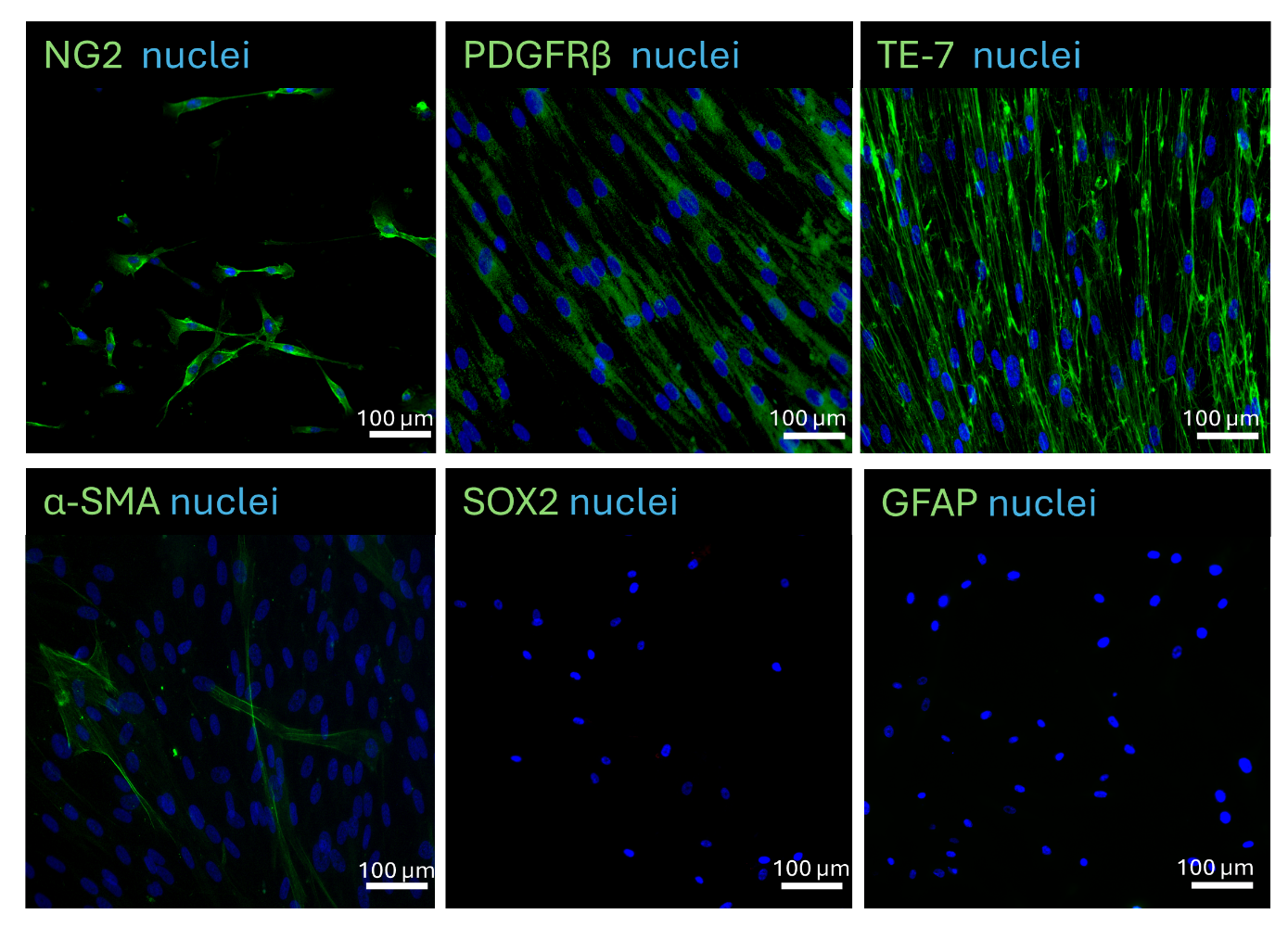


***Supplementary fig. 5: Validation of pericyte characteristics of Human Brain Vascular Pericytes (HBVP)****. HBVP expressed Neural/glial antigen 2 (NG2), platelet derived growth factor receptor beta (PDGFRβ), TE-7, α- smooth muscle actin (α-SMA). Expression of sex determining region Y (SOX2), Glial fibrillary acidic protein-(GFAP) was negative. Expression was detemined by immunucytochemistry, representative images are shown.*

|  | **Culture 46A** | **Culture 80A** | **HBVP** | **U87** | **U251** | **Gelatin only** |  |
| --- | --- | --- | --- | --- | --- | --- | --- |
| **Protein yield (µg)** | 8.63 ± 1.24 | 36.22 ± 3.48 | 127.80 ± 16.84 | 114.38 ± 4.42 | Not detectable | Not detectable |  |

***Supplementary table 1:*** ***Protein yields from cell-derived 3D matrices*** ***produced by various cell types.*** *Numbers are means from four biological replicates ± standard deviations. Protein concentrations in U251 and gelatin samples were under the limit of detection.*
